# Prediction of lipomatous soft tissue malignancy on MRI: comparison between machine learning applied to radiomics and deep learning

**DOI:** 10.1186/s41747-022-00295-9

**Published:** 2022-09-08

**Authors:** Guillaume Fradet, Reina Ayde, Hugo Bottois, Mohamed El Harchaoui, Wassef Khaled, Jean-Luc Drapé, Frank Pilleul, Amine Bouhamama, Olivier Beuf, Benjamin Leporq

**Affiliations:** 1 Capgemini Engineering, Paris, France; 2 Service de Radiologie B, Groupe Hospitalier Cochin, AP-HP Centre, Université de Paris, Paris, France; 3 Université Lyon, INSA-Lyon, Université Claude Bernard Lyon 1, UJM-Saint Etienne, CNRS, Inserm, CREATIS UMR 5220 U1206, Villeurbanne, France; 4 Department of Radiology, Centre de lutte contre le cancer Léon Bérard, Lyon, France

**Keywords:** Artificial intelligence, Machine learning, Magnetic resonance imaging, Deep learning, Soft tissue neoplasms

## Abstract

**Objectives:**

Malignancy of lipomatous soft-tissue tumours diagnosis is suspected on magnetic resonance imaging (MRI) and requires a biopsy. The aim of this study is to compare the performances of MRI radiomic machine learning (ML) analysis with deep learning (DL) to predict malignancy in patients with lipomas oratypical lipomatous tumours.

**Methods:**

Cohort include 145 patients affected by lipomatous soft tissue tumours with histology and fat-suppressed gadolinium contrast-enhanced T1-weighted MRI pulse sequence. Images were collected between 2010 and 2019 over 78 centres with non-uniform protocols (three different magnetic field strengths (1.0, 1.5 and 3.0 T) on 16 MR systems commercialised by four vendors (General Electric, Siemens, Philips, Toshiba)).

Two approaches have been compared: (i) ML from radiomic features with and without batch correction; and (ii) DL from images. Performances were assessed using 10 cross-validation folds from a test set and next in external validation data.

**Results:**

The best DL model was obtained using ResNet50 (resulting into an area under the curve (AUC) of 0.87 ± 0.11 (95% CI 0.65−1). For ML/radiomics, performances reached AUCs equal to 0.83 ± 0.12 (95% CI 0.59−1) and 0.99 ± 0.02 (95% CI 0.95−1) on test cohort using gradient boosting without and with batch effect correction, respectively. On the external cohort, the AUC of the gradient boosting model was equal to 0.80 and for an optimised decision threshold sensitivity and specificity were equal to 100% and 32% respectively.

**Conclusions:**

In this context of limited observations, batch-effect corrected ML/radiomics approaches outperformed DL-based models.

**Supplementary Information:**

The online version contains supplementary material available at 10.1186/s41747-022-00295-9.

## Key points

- Machine learning (ML) applied to magnetic resonance imaging (MRI) radiomics could help to characterise malignancy of lipomatous soft tissue tumours.
- ML/radiomics analysis outperformed DL for the benign/malignant differentiation of lipomatous soft tissue tumours on MRI in a data-limited context.
- Statistical harmonisation using batch effect correction (ComBat method) improved performances when heterogeneous, multicentre data are used.

## Background

Lipomatous soft tissue tumours are a very common neoplasm stemming from fat cells [1]. These tumours are divided into several subgroups, but most of them being benign and referred as lipoma, while rare malignant tumours are referred as liposarcomas [1]. In practice, lipoma and high-grade liposarcoma are easily distinguishable using magnetic resonance imaging (MRI) [2, 3]. Unfortunately, some low-grade liposarcomas subtype called atypical lipomatous tumours (ALTs) representing about 40 to 45% of liposarcomas have overlapping MRI characteristic and are highly similar to lipomas [2–5]. The differential diagnosis between lipomas and ALTs is essential for therapeutic strategy and is based on histology after tissue biopsy. Lipomas are removed by marginal excision if it provides discomfort or pain to the patient, while liposarcomas must be removed by wide margin resection [1, 2]. However, taking into account the time-consuming, financia and invasive burden of biopsy, there is a medical need for providing non-invasive methods. In addition, benign mesenchymal tumours outnumber liposarcomas by a factor of at least 100, most of these biopsies could be avoided.

Radiomics is a recent field of medical imaging analysis in cancer [5, 6]. It consists to convert medical images into mineable and high-dimensional quantitative data (referred as radiomics) using mathematic descriptors. Then, radiomics are used to train machine learning (ML) algorithms to predict an outcome such as malignancy [7]. In parallel, deep learning (DL), based on the use of convolutional neural networks (CNNs), is emerging as a promising field due to its capacity for image classification [8]. However, CNNs often required training on a huge dataset to be accurate.

The aim of this study is to compare MRI radiomics/ML analysis with DL to predict malignancy in patients with lipomatous soft tissue tumours (ALTs versus lipomas).

## Methods

### Patient cohorts

Our institutional review board approved this retrospective study and the requirement to obtain informed consent was waived. The training set was extracted from a labelled database of the radiology department of comprehensive cancer centre Léon Berard. This database is recording patients with lipomatous soft tissue tumours whose histology and fat-suppressed gadolinium contrast-enhanced T1-weighted MRI sequences were available. From December 2010 to January 2018, a total of 85 patients were included (40 with lipomas and 45 with ALTs). Images were collected from 43 different centres with non-uniform protocols and centralised in the Picture Archiving and Communication System of our institution. Acquisitions were performed at three different magnetic field strengths (1.0, 1.5 and 3.0 T) on 16 MR systems commercialised by four vendors (General Electric, Siemens, Philips, Toshiba).

The validation cohort was extracted from a labelled database of the radiology department of CHU Cochin including patients, from July 2012 to July 2019 with lipomatous soft tissue tumours whose histology and MRI scans were available. This cohort included 60 patients (28 with lipomas and 32 with ALTs) with a fat-suppressed gadolinium contrast-enhanced T1-weighted pulse sequence. Images were collected from 35 different centres with non-uniform protocols and centralised in the Picture Archiving and Communication System of our institution. Acquisitions were performed at two different fields (1.5 and 3.0 T) on fifteen MRI systems commercialised by four vendors. For both training cohort and external validation cohort the most commonly used contrast agent was the Dotarem (Guerbet, Villepinte, France) with a dose of 0.2 mL.kg^−1^. Population characteristics for both training and validation set are provided in Table 1.

**Table 1:** Demographic and clinical information for both training and validation set used in this study

	Training set	Validation set
Data size (number of cases)	85 (40 lipomas, 45 ALTs)	60 (28 lipomas, 32 ALTs)
Age (years, mean ± SD)	60.7 ± 11.1	58.0 ± 10.2
Sex	45 females, 40 males	27 females, 33 males
Tumour volume (cm^3^, mean ± SD [range])	371 ± 578 [5.3–3690]	265 ± 314 [3.7–1266]
Tumour major axis length (cm, mean ± SD [range])	9.58 ± 4.76 [2.4–29.4]	9.46 ± 4.83 [2.1–22.7]
Tumour location (number, percentage)		
Lower limbs	57, 67.1%	41, 68.3%
Upper limbs	12, 14.1%	12, 20.0%
Abdomen	7, 8.3%	1, 1.7 %
Torso	9, 10.7%	6, 10.0%

*ALTs* atypical lipomatous tumours, *Range* minimum−maximum values, *SD* standard deviation

### Lesion segmentation

Images were automatically loaded in in-house software developed on Matlab R2019a (The MathWorks, Natick, USA). The tumour was manually segmented in three dimensions, slice-by-slice, by an experienced radiographer with a 19-year experience in MRI and segmentations were reviewed by a radiologist with a 13-year experiences in MRI, using the fat-suppressed gadolinium contrast-enhanced T1-weighted acquisition.

### Reference standard

Data were labelled as malignant or benign based on histopathology using Murine Double Minute 2 (MDM2) gene amplification by fluorescence in situ hybridisation (FISH).

### Radiomics feature extraction

Radiomics features included size, contour and region-based shape features, intensity distribution (or global low-order texture) features, image domain high-order texture features and spatial-frequency textures features. Size and shape features were directly extracted from the binary masks. It included region and edges-based conventional metric. Intensity distribution features were extracted from masked MR images without normalisation or filtering of voxel intensities and from the histogram built with 256 bins. Before the extraction of texture features, voxels were resampled to be isotropic using an affine transformation and a nearest-neighbour interpolation and discretised in a smaller number of grey levels. This operation was performed using an equal probability algorithm to define decision thresholds in the volume such as the number of voxels for a given reconstructed level is the same in the quantised volume for all grey levels. Images were discretised in 8, 16, 24, 32, 40, 48 and 64 grey levels and for each level four matrix were built: grey-level co-occurrence matrix (*n* = 21); grey-level run length matrix (*n* = 13); grey-level size zone matrix) (*n* = 13); and neighbourhood grey tone difference matrix (*n* = 5). From them, characteristics were extracted.

Frequency domain-based texture features were extracted from the Gabor filters responses. Grey-level co-occurrence matrix and grey-level run length matrix were computed for four directions (0°, 45°, 90° and 135°) with an offset of 1 pixel. For grey-level size zone matrix and neighbourhood grey tone difference matrix, a 26-pixel connectivity were used. For Gabor filtering, 5 scales, 6 orientations and a minimal wavelength of 3 were used. Radiomic features computation was achieved according to the image biomarker standardisation initiative, IBSI [9].

Overall, 92 radiomics features were extracted.

### Models training

#### Deep learning on images

MR images were preprocessed with N4 Bias Field Correction [10] algorithm to correct low frequency intensity. Then, the intensities were normalised were normalised using the *Z*-score such as *I*_new_ = (*I*−μ)/σ where μ and σ are the mean and standard deviation of the intensities. Regarding the low number of samples and the complexity of images acquired on different body region, we choose to focus on a classification model only based on the tumour. MRI slices were cropped around the tumours with respect to the masks and resized to a unique matrix size (224 × 224 pixels).

We compared three CNN-based approaches: (i) a custom CNN learned from scratch (global architecture is described in Additional file 1 below); (ii) the fine-tuning of a pretrained ResNet model; and finally (iii) an XGBoost classifier based on a CNN feature extraction. Python *Keras* API with *TensorFlow* [11] backend was used to implement the different CNNs. First, we create a CNN from scratch with a simple architecture containing three blocks including a two-dimensional convolution, a batch normalisation, a ReLU activation, a max pooling and a dropout. After the three blocks, the tensor was flattened and followed by a fully connected layer of 32 units, activated by ReLU. A final dropout was placed before the last layer, composed of a single neuron activated by the sigmoid function to output the probability of malignancy. To augment the size of the dataset, we applied some small transformations on the images (flipped, zoomed, rotated and shifted).

Second, we used transfer learning starting from a ResNet50 model pretrained on ImageNet [12]. The last layers specific to the classification on ImageNet were removed to add a two-dimensional global average pooling giving a flat shape of 2048 features, followed by one or more blocks composed of a fully connected layer, a batch normalisation, a ReLU activation and a dropout. Since the three-dimensional dataset contained more malignant slices than benign ones, we added a *class weight* when fitting the model, to give more importance to each benign observation in the loss function.

As before, the final layer was a single unit, activated by the sigmoid function. We fine-tuned the model by freezing the pretrained part of the network such that only our new top layers could update their weights and biases. The network was trained this way during a few epochs. Then, the last block of the pre-trained part was unfrozen, and trained with a small learning rate. Importantly, images were preprocessed to fit the ResNet50 requirement.

We tested this protocol with training set only tested on external validation cohort and by merging all our data (training and external validation cohort set) over cross-validation (see further in “Models evaluation and statistical analysis” section). Third, we used the ResNet50 to extract features from images, and used these features as inputs to train an XGBoost (eXtreme Gradient Boosting) classifier.

#### Classifier on radiomic data

Since images were acquired on multisite with different MRI acquisition protocols, a stage of harmonisation is necessary to remove the batch effect introduced by technical heterogeneity on radiomic data. Therefore, we apply the ComBat algorithm, a popular batch effect correction tool [13]. Fat signal suppression technique (fat-water decomposition versus fat saturation) having a visible impact on images and being a common source of acquisition protocol difference in clinical routine, we choose this criterion for the batch effect correction.

Four different classifiers from Python *Scikit-learn* [10] were optimised and evaluated: logistic regression (LR), support vector machine (SVM), random forest (RF) and gradient boosting (GB). These classifiers were trained from radiomics with and without batch effect correction for comparison purpose. Each model was fine-tuned with the best hyperparameters for each dataset. For SVM and LR, a preprocessing step was learned on the training set to normalise the features to have zero mean and unit variance. For RF and GB classifier, no standardisation was applied on the features, as it has no effect on decision trees.

### Models evaluation and statistical analysis

Classifiers performances were compared using k-folds cross validation (*k* = 10) on both radiomic and images data. Mean and standard deviation of the area under the curve (AUC) at receiver operating characteristics analysis, sensitivity, specificity, were computed over the 10-folds from the test set (from the training data). Then, we inferred this model on the external validation dataset. For the deep learning approach, multiples slices from the same patient remained in identical fold so that the network could not be learned and tested on two different slices coming from the same patient.

Comparison of model diagnosis performances was achieved by comparing the AUCs from the validation set using the DeLong’s test [14]. Comparisons were done: (i) between the radiomics models data (LR, SVM, RF and GB) trained from harmonised and non-harmonised data; and (ii) between ResNet50 and radiomics model trained from harmonised data.

Sensitivity and specificity comparisons on the training cohort were performed using *χ*^2^ and McNemar test over the 10 cross-validation folds. A *p* value lower than 0.05 was considered as significant.

## Results

### Test cohort

CNN learned from scratch did not succeed to generalise on the test set and result to poor diagnosis performances (AUC 0.53 ± 0.09, mean ± standard deviation). We obtained an AUC of 0.80 ± 0.11 for ResNet50 and of 0.78 ± 0.13 for XGboost trained with CNN features, respectively. Best performances were obtained from batch-corrected radiomic data (AUC 0.99 ± 0.02) compared to non-corrected data with a GB model (AUC 0.83 ± 0.12). Detailed results are provided in Table 2.

**Table 2:** Sensitivity, specificity, AUC (mean ± standard deviation) obtained for each dataset (images, radiomics with and without batch effect normalisation) and classifier combination over cross-validation obtained using 10 cross-validation folds on the test cohort

Dataset	Classifier	Sensitivity (%)	Specificity (%)	AUC
Images	CNN	90 ± 0.21	10 ± 0.10	0.53 ± 0.09
	ResNet50	81.9 ± 0.06	57.7 ± 0.07	0.8 ± 0.11
	FE + XGB	80.3 ± 0.12	57 ± 0.11	0.78 ± 0.13
Radiomics	LR	77 ± 0.19	62.5 ± 0.24	0.84 ± 0.12
	SVM	75 ± 0.14	62.5 ± 0.26	0.83 ± 0.43
	RF	84 ± 0.01	62.5 ± 0.12	0.79 ± 0.65
	GB	72.5 ± 0.05	72.5 ± 0.16	0.83 ± 0.12
Radiomics with batch correction	LR	70 ± 0.22	77.5 ± 0.31	0.86 ± 0.13
	SVM	75 ± 0.17	70 ± 0.25	0.82 ± 0.15
	RF	100 ± 0.18	92.5 ± 0.33	0.96 ± 0.04
	GB	98 ± 0.20	87.5 ± 0.13	0.99 ± 0.02

*AUC* area under the curve, *CNN* convolutional neural networks, *FE* feature extraction, *XGB* Xgboost, *GB* gradient boosting, *LR* logistic regression, *RF* random forest, *SVM* support vector machine

### External validation cohort

We tested all previously trained models on the external validation cohort.

We noticed a decrease performance of ResNet model on validation cohort compared to test cohort used during training (from AUC = 0.80 ± 0.11 to AUC = 0.64 respectively). We did not obtain better performance (AUC 0.74 ± 0.12, sensitivity 80%, specificity 53%) by adding patients from validation cohort in the training set (Table 3).

**Table 3:** Sensitivity, specificity and AUC obtained from previously trained models inferring on the external validation cohort with corresponding data correction

Dataset	Classifier	Sensitivity (%)	Specificity (%)	AUC
Images	ResNet50	92	24	0.64
Radiomics	LR	1	0	0.50
	SVM	70	32	0.47
	RF	64	68	0.71
	GB	67	64	0.70
Radiomics with batch correction	LR	1	0.07	0.54
	SVM	47	57	0.48
	RF	53	86	0.75
	GB	97	61	0.80

*AUC* area under the curve, *CNN* convolutional neural networks, *FE* feature extraction, *XGB* Xgboost, *GB* gradient boosting, *LR* logistic regression, *RF* random forest, *SVM* support vector machine

**Fig. 1:**
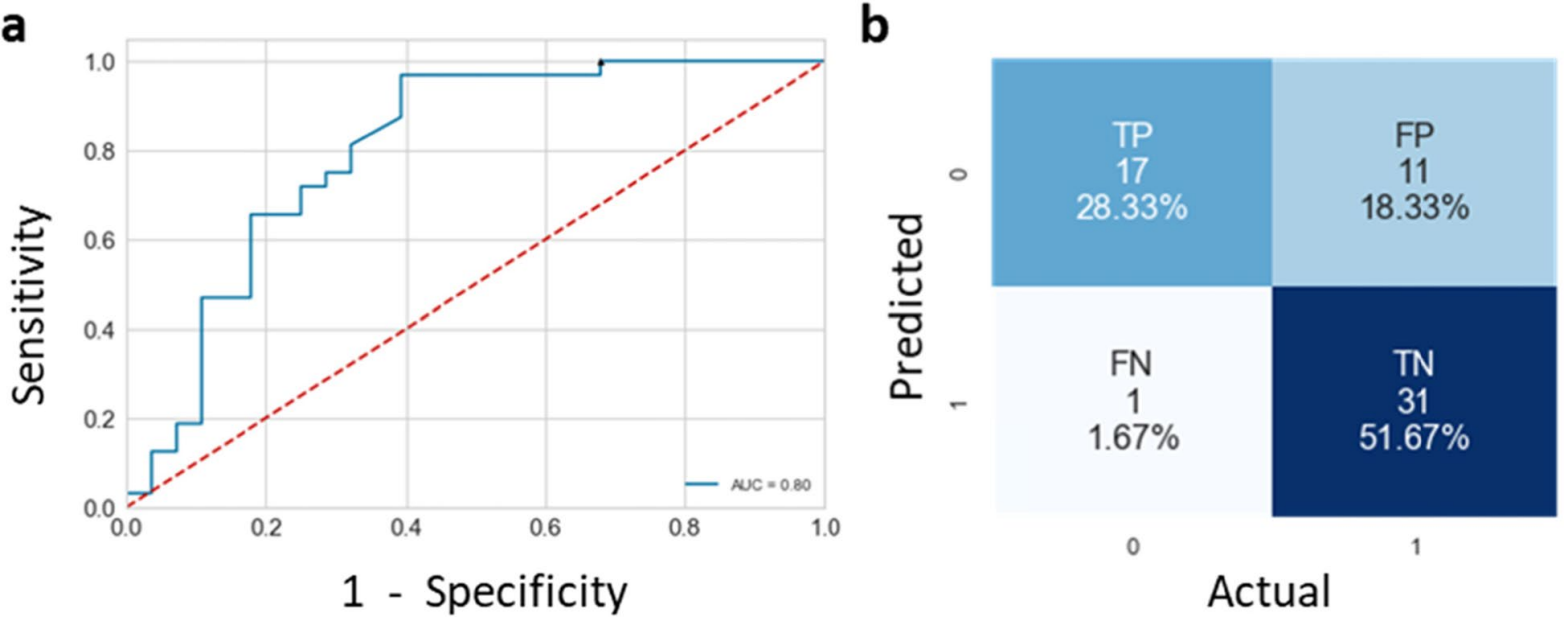
Receiver operating characteristics curve (**a**) and confusion matrix (**b**) from gradient boosting models on batch corrected validation data

**Fig. 2:**
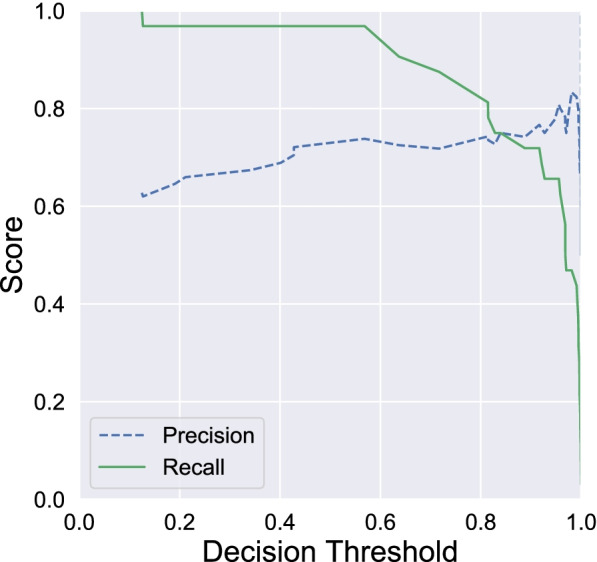
Precision and sensitivity score of gradient boosting models on validation data with different decision threshold

For the radiomic approach models trained with batch-corrected data globally resulted in a better performance than those trained with non-corrected data on external validation cohort, but no statistical differences have been found. Our best model obtained an AUC of 0.80 with a high sensitivity of 97% and a specificity of 61% (Fig. 1, Table 2). In spite of specificity loss, we optimised the GB decision threshold to increase sensitivity. We reach 100% of sensitivity and 32% of specificity by decreasing standard decision threshold from 0.5 to 0.1 (Fig. 2 and Table 4).

**Table 4:** Table containing computed metrics (sensitivity, specificity and F1 score) obtained from validation data with gradient boosting models with decision threshold of 0.5 or 0.1

Threshold	Class	Specificity (%)	Sensitivity (%)	F1 score
**0.5**	Malignant	74	97	84
	Benign	94	61	74
**0.1**	Malignant	63	100	77
	Benign	100	32	49

**Fig. 3:**
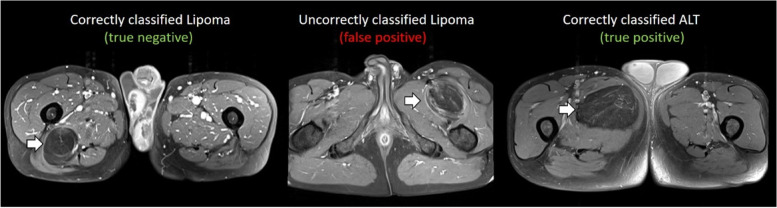
Examples of true negative, false positive and true positive on external data given by the predictive model trained from ComBat-harmonised radiomics features with the gradient boosting classifier

Examples of MRI from patients obtaining true negative, false positive and true positive with gradient boosting classifier trained on combat-harmonised radiomics are show in Fig. 3.

### AUC and metrics comparisons

No significant differences were found between radiomics model trained from harmonised and non-harmonised data. DeLong’s test *p* values were equal to 1.0, 0.33, 0.96 and 0.22 for the LR, SVM, RF and GB models respectively. However, the “harmonised” GB model had a better sensitivity and specificity compared to non-harmonised counterpart. We selected the GB model trained on harmonised performance for decision threshold optimisation. However, we did not find significant differences between harmonised and non-harmonised radiomic trained GB models for sensitivity and specificity using *χ*^2^ (*p* = 0.550 and *p* = 0.414 respectively) over the 10 cross-validation folds during training with test cohort. Significant differences were found between ResNet50 model and between radiomics model trained from harmonised data (*p* < 0.001 for all).

## Discussion

In this study, we have shown that ML from MRI radiomics could be relevant to classify patient with lipoma or ALTs and therefore to potentially reduce the number of biopsies. The results also demonstrate the need to correct radiomics data for batch effect linked to heterogeneity in the MRI acquisition protocol. In our context of limited observations, batch corrected radiomic-based models outperformed the CNN approaches.

Using radiomic features, and traditional ML classifiers, we obtained a sensitivity of 100% and a specificity of 32% on an external validation cohort. It indicates that 32% of biopsies could be avoided for negative patient.

This work need to be confirmed on a larger study cohort. As previously demonstrated [15, 16], our results suggest that batch correction on radiomic data using ComBat method is useful with heterogeneous data, due to variability in MRI acquisition protocols from different imaging departments and hardware capabilities are used. Another work reported similar performances to diagnose well-differentiated lipomatous tumours: from radiomics derived from unenhanced T1- and T2-weighted MRI sequences, Vos et al. [17] obtained an AUROC equal to 0.89, however these results were not validated in an external cohort. From T1-weighted MRI radiomics, Malinauskaite et al. [18] obtained higher performances (AUC 0.926) but the volume of data was small (*n* = 38) and no validation on external validation set was proposed. Pressney et al. [19] have proposed a composite score (AUC 0.80) built from a multivariate analysis combining qualitative imaging features and texture features derived from T-weighted and proton density-weighted images However, no cross-validation techniques was performed and the size of data was relatively small (*n* = 60). To the best of our knowledge, the present work is the first reporting results from external validation data, a mandatory issue to identify harmonisation problem linked to acquisition protocol heterogeneities.

Using directly the images and CNNs was challenging in this context of *domain generalisation* [20], which consists of training a model on multiple source domains, and evaluate its performance on a distinct and unseen target domain. Thus, high heterogeneity in the images from various body regions made the task of generalisation difficult for the CNN.

Unlike radiomics, CNNs do not use quantitative features like tumour size as images had different zoom levels. The CNN performance might have been higher if the MRI slices were set to a unique scale, but we wanted the CNN to find other decision characteristics than the tumour size. In addition, it is more difficult to correct the batch effect with CNNs. Some way of investigation using native images harmonisation from generative adversarial networks could be envisioned in furthers works [21–24].

However, it is important to note that manual tumour segmentation may introduce inherent variability on radiomic features and constitutes a time-consuming task for the radiologist [5]. Segmentation time depends on the number of slices and on the tumour volume. As an example, in this work, average segmentation time was located around five minutes for a range between two to ten minutes. Therefore, further works to substitute this task by automated approaches based on U-Net could be relevant [25]. Another limitation of this study might be the choice of only fat-suppressed gadolinium contrast-enhanced T1-weighted MRI sequences to perform radiomic analysis. Since gadolinium injection increases costs, is not systematically done in routine use, radiomic analysis from unenhanced sequences need to be investigated.

To conclude, radiomic ML analysis outperformed DL-based approach to predict malignancy in lipomatous soft tissue tumours due to the possibility to *a posteriori* correct for acquisition heterogeneity. We probably could obtain better performance with more data as DL approaches are usually very performant but need a lot more data than ML analysis of radiomics data. In addition, it is much harder to generalise classification for tumours located on various organs, due to the high heterogeneity in the images as this is the case with soft-tissue tumours. Manual segmentation is also time-consuming and may introduce variability in radiomics. In a future, DL for classification tasks after generative adversarial networks-based image harmonisation or radiomic analysis after U-Net automated segmentation could help to overcome this issue.

## Supplementary Information

**Additional file 1.** Electronic supplementary material.

## Data Availability

The datasets used and analysed during the current study are available from the corresponding author on reasonable request.

## Abbreviations

ALTs: Atypical lipomatous tumours
AUC: Area under the curve
CNN: Convolutional neural networks
DL: Deep learning
GB: Gradient boosting
LR: Logistic regression
ML: Machine learning
MRI: Magnetic resonance imaging
RF: Random forest
SVM: Support vector machine

## References

[1] Jebastin JAS, Perry KD, Chitale DA (2020). Atypical lipomatous tumor/well-differentiated liposarcoma with features mimicking spindle cell lipoma. Int J of Surg.

[2] Knebel C, Lenze U, Pohlig F (2017). Prognostic factors and outcome of liposarcoma patients: a retrospective evaluation over 15 years. BMC Cancer.

[3] Brisson M, Kashima T, Delaney D (2013). MRI characteristics of lipoma and atypical lipomatous tumor/well- differentiated liposarcoma: retrospective comparison with histology and MDM2 gene amplification. Skeletal Radiol.

[4] Leporq B, Bouhamama A, Pilleul F (2020). MRI-based radiomics to predict lipomatous soft tissue tumors malignancy: a pilot study. Cancer Imaging.

[5] Fletcher C, Unni K, Mertens F (2002) Pathology and genetics of tumours of soft tissue and bone. iarc

[6] Gillies RJ, Kinahan PE, Hricak H (2016). Radiomics: Images are more than pictures, they are data. Radiology.

[7] Aerts HJWL, Velazquez ER, Leijenaar RTH (2014). Decoding tumour phenotype by noninvasive imaging using a quantitative radiomics approach. Nature Commun.

[8] Lundervold AS, Lundervold A (2019). An overview of deep learning in medical imaging focusing on MRI. Z Med Phys.

[9] Zwanenburg A, Vallières M, Abdalah MA (2020). The image biomarker standardization initiative: standardized quantitative radiomics for high-throughput image-based phenotyping. Radiology.

[10] Tustison NJ, Avants BB, Cook PA (2010). N4ITK: Improved N3 bias correction. IEEE Trans Med Imaging.

[11] Martin A, Ashish A, Paul B, et al (2015) TensorFlow: Large-Scale Machine Learning on Heterogeneous Systems. arXiv preprint arXiv:1603.04467. 10.48550/arXiv.1603.04467

[12] Krizhevsky A, Sutskever I, Hinton GE (2012). ImageNet Classification with Deep Convolutional Neural Networks. Adv Neural Inf Process Syst..

[13] Johnson WE, Li C, Rabinovic A (2007). Adjusting batch effects in microarray expression data using empirical Bayes methods. Biostatistics.

[14] DeLong ER, DeLong DM, Clarke-Pearson DL (1988). Comparing the Areas under Two or More Correlated Receiver Operating Characteristic Curves: A Nonparametric Approach. Biometrics.

[15] Orlhac F, Frouin F, Nioche C (2019). Validation of a method to compensate multicenter effects affecting CT radiomics. Radiology.

[16] Orlhac F, Lecler A, Savatovski J (2020). How can we combat multicenter variability in MR radiomics? Validation of a correction procedure. Eur.

[17] Vos M, Starmans MPA, Timbergen MJM (2019). Radiomics approach to distinguish between well differentiated liposarcomas and lipomas on MRI. BJS.

[18] Malinauskaite I, Hofmeister J, Burgermeister S (2020). Radiomics and Machine Learning Differentiate Soft-Tissue Lipoma and Liposarcoma Better than Musculoskeletal Radiologists. Sarcoma.

[19] Pressney I, Khoo M, Endozo R (2020). Pilot study to differentiate lipoma from atypical lipomatous tumour/well-differentiated liposarcoma using MR radiomics-based texture analysis. Skeletal Radiol.

[20] Wang J, Lan C, Liu C et al (2021) Generalizing to unseen domains: a survey on domain generalization. IEEE Trans Knowl Data Eng. 10.1109/TKDE.2022.3178128

[21] Armanious K, Jiang C, Fischer M (2020). MedGAN: Medical image translation using GANs. Comput. Med. Imaging Graph..

[22] Bowles C, Chen L, Guerrero R, et al (2018) GAN augmentation: augmenting training data using generative adversarial networks. arXiv preprint arXiv:1810.10863. 10.48550/arXiv.1810.10863

[23] Karras T, Aila T, Laine S, Lehtinen J (2017) Progressive growing of gans for improved quality, stability, and variation. arXiv preprint arXiv:1710.1019624. 10.48550/arXiv.1710.10196

[24] Nie D, Trullo R, Lian J (2018). Medical image synthesis with deep convolutional adversarial networks. IEEE Trans. Biomed. Eng.

[25] Ronneberger O, Fischer P, Brox T (2015). U-net: Convolutional networks for biomedical image segmentation. In: Lecture Notes in Computer Science (including subseries Lecture Notes in Artificial Intelligence and Lecture Notes in Bioinformatics). Springer Verlag.

