# Supplementary material for: Prediction of lipomatous soft tissue malignancy on MRI: comparison between machine learning applied to radiomics and deep learning
Source: Eur Radiol Exp. 2022 Sep 8;6:41. doi: 10.1186/s41747-022-00295-9 (PMC9452614; doi:10.1186/s41747-022-00295-9)
Supplement: Supplementary file 1 — Additional file 1. Electronic supplementary material. [file 41747_2022_295_MOESM1_ESM.pdf]

ELECTRONIC SUPPLEMENTARY MATERIAL

Prediction of lipomatous soft tissue malignancy on MRI: comparison between machine learning applied to radiomics and deep learning

Appendix

With the Keras API, we developed a convolutional neural network (CNN) where we defined each layer. Because the model was learnt from scratch with a small amount of samples, we kept the architecture simple, without too many parameters. The input images were rescaled to a unique size (128 × 128), and kept as grayscale (one channel). The input shape was therefore (*batch,* 128*,* 128*,* 1). The pixel intensities were normalized between 0 and 1. Three blocks containing a two-dimensional convolution, a batch normalization, a ReLU activation, a max pooling and a dropout were repeated. All the convolutions had a kernel size of 3 × 3, a padding such that size of the input and output remained the same, and the weights initialized randomly by the Glorot uniform initializer. The first convolution created 16 feature maps (number of channels), the second convolution outputed 32, while the last one produced 64. The pooling size was 2×2, such that the size was divided by two after each max pooling layer. The dropout rate was fixed to 0.5. After the three blocks, the tensor was flattened and followed by a fully connected layer of 32 units, activated by ReLU. A final dropout was placed before the last layer, composed of a single neuron. The latter was activated by the sigmoid function to output a probability. Adam optimizer was used to minimize the model's loss (binary cross-entropy). The network had approximately 550 000 trainable parameters. To augment the size of the dataset, we applied some small transformations on the images, so that the network could not see the exact same image twice. They were randomly flipped, zoomed, rotated and shifted. We did not used heavy transformation like shear mapping, which would alter the true shape of the tumor.
